# Treatment-Resistant Psychosis in an Adolescent at Risk of Metabolic Syndrome: A Case Report

**DOI:** 10.7759/cureus.116658

**Published:** 2026-09-21

**Authors:** Michael Belkin, Jasmin Grewal

**Affiliations:** 1 Psychiatry and Behavioral Sciences, New York Institute of Technology (NYIT) College of Osteopathic Medicine, Old Westbury, USA; 2 Psychiatry and Behavioral Sciences, Nassau University Medical Center, East Meadow, USA

**Keywords:** child and adolescent psychiatry, clozapine, diabetes and metabolic syndrome, schizophrenia and other psychotic disorders, treatment-resistant psychosis

## Abstract

Management of treatment-resistant psychosis is inherently complex, presenting unique clinical challenges in pediatric populations when metabolic syndrome risk limits pharmacologic options. We report a case of an adolescent with rapid decline and persistent psychotic symptoms despite trials of first-line antipsychotic agents. His refractory clinical course required careful consideration of the relative benefits of other antipsychotic therapies against their potential for significant metabolic adverse effects. Clinical decision-making was further complicated by the need to engage the patient’s mother in consent discussions and shared treatment decisions. This case highlights the clinical challenges of treating pediatric treatment-resistant psychosis while balancing psychiatric stabilization, cardiometabolic risks, and family-centered consent.

## Introduction

Treatment-resistant schizophrenia (TRS) is defined as the persistence of significant symptoms and functional impairment despite at least two adequate trials of different antipsychotics at therapeutic doses for a sufficient duration with confirmed medication adherence [1]. TRS occurs in approximately 24% of patients presenting with first-episode schizophrenia, while pediatric-specific prevalence estimates remain limited [2]. 

In children and adolescents presenting with first-episode schizophrenia, treatment generally begins with a single antipsychotic medication, with risperidone and aripiprazole among the most commonly used first-line agents [3]. Current pediatric guidelines recommend evaluating response after approximately 4-6 weeks at an optimized dose, with consideration of an alternative antipsychotic when there is inadequate response or intolerable adverse effects [4]. In youth whose illness remains inadequately responsive despite sequential adequate trials of two different antipsychotics, initiation of clozapine should be considered for persistent treatment-resistant illness [3].

The metabolic consequences of antipsychotic therapy are an important consideration in pediatric patients, particularly with second-generation antipsychotics (SGAs). Metabolic syndrome is characterized by a clustering of cardiometabolic risk factors. In pediatric patients aged 10 to <16 years, the International Diabetes Federation defines metabolic syndrome as the presence of abdominal obesity, defined as a waist circumference ≥90th percentile, and at least two of the following four criteria: triglycerides ≥150 mg/dL, high-density lipoprotein (HDL) cholesterol <40 mg/dL, systolic blood pressure ≥130 mmHg or diastolic blood pressure ≥85 mmHg, or fasting plasma glucose ≥100 mg/dL or known type 2 diabetes mellitus [5].

While clozapine remains the gold standard for treatment-resistant psychosis, its profound metabolic liability presents a formidable clinical dilemma, particularly when prescribed to pediatric patients who already suffer from pre-existing type 2 diabetes and severe obesity. Balancing psychiatric stabilization against the risk of catastrophic metabolic exacerbation is a major challenge in pediatric psychopharmacology, and data regarding safe pharmacologic mitigation strategies remain scarce. Although glucagon-like peptide-1 (GLP-1) receptor agonists have emerged as effective tools for managing antipsychotic-induced metabolic complications, their concurrent integration into complex pediatric cases requiring clozapine is underexplored. Herein, we present the educational and clinical management of a 15-year-old male patient with treatment-resistant schizoaffective disorder, pre-existing type 2 diabetes, and severe obesity who required clozapine therapy, highlighting a practical multidisciplinary approach using a GLP-1 receptor agonist to mitigate severe cardiometabolic risks in an otherwise prohibitive clinical scenario.

## Case presentation

A 15-year-old male patient with a history of attention-deficit/hyperactivity disorder (ADHD) and recently diagnosed type 2 diabetes mellitus initially presented to an outside hospital with progressive manic and psychotic symptoms. His presentation was characterized by aggression, auditory hallucinations, paranoia, disorganized behaviors and thought processes, insomnia, increased psychomotor activity, and grandiosity. He was subsequently transferred to our psychiatric emergency department as a voluntary admission to the child and adolescent inpatient psychiatric unit.

One week prior to his presentation at our emergency department, he had been discharged from an outside inpatient psychiatric unit, where he had also been hospitalized and discharged several weeks earlier. At the time of that earlier discharge, he was given a provisional diagnosis of schizophrenia versus substance-induced psychosis. Despite reported adherence to chlorpromazine for psychosis and agitation/aggression, lamotrigine for mood stabilization, and methylphenidate for ADHD, as well as outpatient follow-up, his symptoms persisted after discharge. His mother contacted his outpatient provider due to persistent insomnia, who initiated mirtazapine; however, she reported minimal improvement, and the patient discontinued the medication after only several doses, prompting evaluation at our institution.

On initial presentation, his height was 177 cm and weight was 122 kg, corresponding to a body mass index (BMI) of 38.9 kg/m². During the initial evaluation, he was generally cooperative, intermittently tangential, and occasionally disorganized, unable to provide clear and direct responses. Symptoms of insomnia and grandiose delusions were elicited during the initial evaluation. He had reported a history of auditory hallucinations, with the most recent episode occurring shortly before or during his presentation to the emergency department. He initially described these experiences as “noises and voices speaking incoherently.” Upon further exploration, however, he attributed these experiences to “misinterpreting” what others were saying rather than experiencing true auditory hallucinations. 

Collateral information obtained from his mother revealed various manic and psychotic symptoms with a rapid deterioration after his symptoms first emerged approximately seven months earlier. Over the subsequent months, he became increasingly disorganized and internally preoccupied and developed escalating verbal and physical aggression toward her. During this time, he became increasingly isolated from peers and his performance in school declined. He had repeated suspensions from school due to fighting since the onset of his psychiatric symptoms. There was a report of significant marijuana use but no known history of other substance use. He had an unremarkable birth history and met all developmental milestones. He was diagnosed with ADHD in eighth grade and subsequently received an Individualized Education Program (IEP). His family psychiatric history was notable for a sibling with schizoaffective disorder and a father with schizophrenia. 

Medication reconciliation at the time of admission revealed a regimen consisting of chlorpromazine, methylphenidate, lamotrigine, mirtazapine, clonidine, and metformin. Given concern that stimulant therapy could exacerbate his psychotic symptoms, methylphenidate was discontinued. Mirtazapine was also discontinued, while chlorpromazine was gradually cross-tapered to risperidone. Risperidone was selected for treatment of his persistent psychotic symptoms, with consideration given to a reported favorable response to risperidone in a family member. Clonidine and metformin were continued.

His initial response to risperidone was limited. He demonstrated mild improvement in disorganization and displayed little to no aggression. Over the subsequent days, his clinical status remained largely unchanged, and staff frequently observed him talking to himself. During this period, records from his prior psychiatric hospitalization were obtained and reviewed. These records documented sequential treatment with multiple antipsychotic medications, including olanzapine, haloperidol, and chlorpromazine (Table 1). The available records did not consistently document the clinical rationale for individual medication changes or the patient's response to each medication.

**Table 1:** Documented antipsychotic treatment during prior psychiatric hospitalization

Date	Medication Regimen
Day 1	Olanzapine 10 mg twice daily continued; haloperidol 1 mg nightly initiated
Day 7	Olanzapine decreased to 5 mg nightly; haloperidol increased to 2 mg nightly
Day 8	Olanzapine discontinued
Day 11	Haloperidol increased to 5 mg nightly
Day 12	Haloperidol increased to 5 mg twice daily
Day 13	Haloperidol decreased to 2 mg each morning and 5 mg nightly
Day 15	Morning haloperidol discontinued; haloperidol continued at 7 mg nightly
Day 19	Haloperidol discontinued; chlorpromazine 50 mg nightly initiated

Over the subsequent 10 days, he demonstrated no meaningful improvement in his psychotic symptoms and became increasingly paranoid toward peers and staff, and grandiose delusions persisted.

In the context of persistent psychotic symptoms, prior trials of multiple antipsychotic medications, and inadequate response to risperidone during the current hospitalization, the decision was made, in collaboration with his mother, to initiate clozapine and submit a referral for admission to a state psychiatric hospital for ongoing inpatient stabilization and treatment.

Throughout his hospitalization, his weight increased from 122 kg on admission to 129 kg over approximately one month, despite treatment with metformin 1,000 mg twice daily. His blood pressure became elevated, with average measurements of 140s/90s mmHg, raising concerns of metabolic syndrome. Clozapine titration was temporarily suspended due to clinically significant weight gain, elevated blood pressure, and his mother’s declination of consent for continued titration until initiation of a GLP-1 agonist to mitigate clozapine associated weight gain. Insurance authorization barriers delayed initiation of semaglutide, resulting in temporary clozapine titration suspension for approximately 12 days. 

During subsequent clozapine dose escalation, the patient demonstrated a reduction in disorganization and no further aggressive behaviors. Paranoia toward peers resolved, and episodes of internal preoccupation decreased substantially. While he appeared to have a clinically meaningful response to clozapine, further titration to a higher dose was anticipated to optimize therapeutic benefit given persistent intermittent psychotic signs and symptoms. He was accepted and transferred to a state psychiatric hospital for ongoing inpatient stabilization, treatment, and clozapine titration.

## Discussion

This case illustrates the challenges of managing treatment-resistant psychosis in an adolescent, particularly in the setting of pre-existing metabolic disease. Despite exposure to multiple antipsychotic medications across several hospitalizations, the patient continued to experience persistent psychotic symptoms and behavioral deterioration, ultimately prompting initiation of clozapine. His clinical course was further complicated by progressive weight gain and elevated blood pressure measurements, requiring the addition of semaglutide to mitigate the risk of developing metabolic syndrome. This case highlights the therapeutic challenge of balancing the efficacy of clozapine in treatment-resistant psychosis with its substantial metabolic risks, particularly in an adolescent already at elevated cardiometabolic risk.

Although TRS is relatively well characterized in adults, its prevalence, diagnostic criteria, and optimal management in children and adolescents remain less clearly established. The identification of psychotic symptoms in younger patients can be challenging, as developmental factors may complicate the differentiation of normative experiences from clinically significant hallucinations or delusions [6]. The emergence of psychotic symptoms may also be preceded by a prodromal phase characterized by a period of negative symptoms, including progressive cognitive and social decline, resulting in more difficulty identifying a primary psychotic illness among children and adolescents [6]. In our patient, a progressive decline in cognition and behavior preceded the emergence of prominent psychotic symptoms, consistent with this recognized pattern of illness progression.

Treatment of childhood and early adult-onset schizophrenia also remains relatively understudied, with limited high-quality evidence to guide pharmacologic management [7]. SGAs are commonly used in pediatric patients because of their generally lower risk of extrapyramidal symptoms compared with first-generation antipsychotics. However, evidence regarding comparative efficacy among antipsychotic agents in youth remains limited. The Treatment of Early-Onset Schizophrenia Spectrum Disorders (TEOSS) study found no significant differences in overall symptom improvement among olanzapine, risperidone, and molindone, while notable differences in adverse effect profiles were observed [8]. These findings highlight the importance of considering both efficacy and side-effect profiles when selecting antipsychotic therapy in pediatric patients. In particular, the metabolic adverse effects associated with several SGAs warrant careful consideration, especially in patients with pre-existing obesity or metabolic disease.

Clozapine remains a mainstay of treatment for TRS and has demonstrated efficacy in childhood- and adolescent-onset schizophrenia [9]. Although large studies evaluating the efficacy and safety of clozapine in pediatric populations remain limited, available evidence suggests that clozapine may provide greater efficacy than other antipsychotic medications in treatment-resistant illness, with benefits demonstrated in both short- and long-term treatment. A systematic review of clozapine use in childhood- and adolescent-onset schizophrenia found that improvements in overall symptoms were observed with short-term treatment and were maintained during long-term follow-up, with some studies also reporting favorable clinical response and shorter hospital stays [10]. However, clozapine was associated with a substantial adverse-effect burden, including sedation, hypersalivation, constipation, neutropenia, weight gain, and metabolic abnormalities [10].

The metabolic effects of clozapine are of particular concern in patients with pre-existing cardiometabolic disease. In the reviewed pediatric literature, weight gain was reported in up to 64% of patients and metabolic abnormalities in 8-22%, while treatment-emergent diabetes was reported less frequently [11]. This is particularly relevant to our patient, who had pre-existing type 2 diabetes and obesity prior to clozapine initiation and subsequently experienced further weight gain during hospitalization.

Particular attention should be given to the metabolic effects of antipsychotic treatment in antipsychotic-naïve children and adolescents. Short-term antipsychotic exposure in this population has been associated with rapid and significant weight gain [11]. In a prospective study by Correll et al., antipsychotic-naïve youth aged 4-19 years were followed for approximately 12 weeks after initiating treatment with one of four SGAs [11]. The authors found significant weight gain across all four medications, with olanzapine associated with the greatest increase in weight. Furthermore, metabolic effects varied among medications, with olanzapine demonstrating the most pronounced adverse effects on glucose and lipid parameters, while quetiapine and risperidone were primarily associated with increases in triglycerides [11]. These findings underscore the importance of careful metabolic monitoring when initiating antipsychotic therapy in children and adolescents.

When starting patients on antipsychotics, clinicians should consider strategies to minimize the metabolic risks associated with treatment. Metformin is one of the most extensively studied adjunctive medications for the prevention and treatment of antipsychotic-associated weight gain. It primarily acts by enhancing insulin action and reducing hepatic glucose production [12]. Metformin has also been shown to reduce appetite and caloric intake, which may contribute to its effects on body weight [13]. Large-scale meta-analyses have demonstrated that adjunctive metformin use in patients receiving antipsychotics results in reduced weight gain and improvements in several metabolic parameters compared with placebo [14]. However, the magnitude of weight loss associated with metformin is generally modest, and not all patients achieve clinically meaningful weight reduction.

When metformin alone is insufficient to mitigate antipsychotic-associated weight gain or when significant metabolic complications persist, additional pharmacologic strategies may be considered. GLP-1 receptor agonists are a relatively newer approach, and evidence regarding their use for antipsychotic-associated weight gain remains limited. However, emerging studies have demonstrated promising results. In a 2017 randomized clinical trial, 103 patients with schizophrenia-spectrum disorders receiving clozapine or olanzapine were randomized to receive either liraglutide or placebo for 16 weeks [15]. Compared with placebo, liraglutide treatment resulted in significant improvements in glucose tolerance, body weight, waist circumference, and visceral fat [15]. These findings may provide preliminary support for the potential role of GLP-1 receptor agonists in managing antipsychotic-associated metabolic complications. These findings are relevant to our patient, who experienced progressive weight gain, raising concerns for metabolic complications despite treatment with metformin, ultimately prompting initiation of semaglutide. 

## Conclusions

This case highlights the challenges of managing TRS in an adolescent and the subsequent use of clozapine in the setting of significant metabolic complications. TRS in adolescents presents challenges in both medication selection and management of adverse effects with guardian consent, particularly in patients with pre-existing metabolic diseases. Clozapine remains an important treatment option for refractory illness, but its metabolic adverse effects require careful monitoring and management. Given the complexity of balancing the potential benefits and risks of pharmacotherapy, treatment decisions should incorporate an individualized assessment of the psychiatric benefits of the antipsychotic relative to its cardiometabolic risks. A patient-centered approach to ongoing metabolic monitoring and risk mitigation is essential to support both psychiatric stability and long-term physical health. Adjunctive therapies such as metformin and GLP-1 receptor agonists may provide useful strategies for mitigating antipsychotic-associated metabolic complications when lifestyle interventions alone are insufficient, though further rigorous prospective studies are needed to establish their safety, long-term efficacy, and optimal implementation in pediatric populations.
